# Nanomaterial Enhanced PVDF Mixed Matrix Membranes for Microfluidic Electrochemical Desalination

**DOI:** 10.3390/membranes16020062

**Published:** 2026-02-02

**Authors:** Haya Taleb, Gopal Venkatesh, Sofian Kanan, Raed Hashaikeh, Nidal Hilal, Naif Darwish

**Affiliations:** 1 Department of Chemical and Biological Engineering, College of Engineering, American University of Sharjah, Sharjah P.O. Box 26666, United Arab Emirates; 2 Department of Biology, Chemistry & Environmental Sciences, College of Arts and Sciences, American University of Sharjah, Sharjah P.O. Box 26666, United Arab Emirates; 3 NYUAD Water Research Centre, New York University, Abu Dhabi Campus, Abu Dhabi P.O. Box 129188, United Arab Emirates

**Keywords:** PVDF-based membranes, desalination, ion removal efficiency, specific energy

## Abstract

This work provides a systematic experimental study for the electrochemical desalination of saline water using an electrospun permselective polyvinylidene difluoride (PVDF) membrane. Several nano additives were initially screened during membrane development; however, only the materials that demonstrated stable dispersion, reproducible membrane formation, and consistent electrochemical behaviour, namely graphene oxide (GO) and carbon nanotubes (CNTs) were selected for full analysis in this study. Accordingly, the study focuses on pure PVDF, PVDF/GO, and PVDF/CNTs membranes integrated with an alternating Ag/AgCl electrode system. The silver electrode is prepared by spray-coating of silver nanoparticles on high surface carbon cloth, whereas the AgCl electrode was prepared electrochemically from the Ag electrode using a three-electrode electrochemical cell. The electrochemical behaviour of various modified electrodes (bare carbon cloth, Ag/carbon cloth, Ag/nafion/carbon black/PVDF, and Ag/nafion/carbon cloth) was evaluated using cyclic voltammetry (CV), electrochemical impedance spectroscopy (EIS), and X-Ray Diffraction (XRD). The electrode prepared using Nafion and PVDF as binders with carbon black as conductive additive exhibited the highest current response and lowest charge-transfer resistance. When coupled with this optimized electrode, the PVDF/GO membrane delivered the best desalination performance, achieving an ion removal efficiency of 68%, a salt adsorption capacity (SAC) of 775.40 mg/g, and a specific energy consumption (SEC) of 16.17 kJ/mole values superior to those reported in the literature.

## 1. Introduction

The field of microfluidic electrochemical desalination has arisen as a ray of hope in the quest for sustainable water management, with the potential to completely transform conventional methods of desalination [1]. Combining microfluidics, electrochemistry, membrane science, and desalination into what’s called microfluidic electrochemical desalination offers a novel approach to address the increasingly pressing problem of water scarcity [2]. This technology derives importance from its capacity to combine the accuracy of microfluidic systems with the laws of electrochemistry, leading to effective, scalable, and ecologically friendly desalination processes [3]. With microfluidic systems, the manipulation of small amounts of liquids is possible, resulting in improved mass transfer and better salt removal efficacy [4]. The integration of microfluidic desalination with electrochemical techniques is expected to improve the selectivity of ion transport. The high costs associated with membranes and electrode systems remain a huge obstacle for large-scale applications. The development of cost-effective membrane–electrode assembly, therefore, becomes of urgent priority [5,6].

Most recently, researchers have focused on the combination of nanomaterials, ion-exchange membranes, and customized electrodes to boost the ion removal efficiency and scalability of the desalination systems [7]. The use of small and portable devices will open the door to reach proper scalability levels and offer water to places where water resources are limited. Although microfluidic electrochemical desalination seems a promising process towards the issue of water scarcity, various difficulties are still encountered in terms of costs and scalability needs. These obstacles should be comprehended accurately to guide future research innovations [8]. Overcoming these challenges will require interdisciplinary collaboration and the development of cost-effective and robust materials. Additionally, the optimization of energy consumption within microfluidic systems is a crucial factor to ensure the sustainability of the technology. Also, the following issues present obstacles towards large-scale commercial operations: (i) high manufacturing and material costs of commercially available ion-exchange membranes, (ii) membrane durability and replacement frequency, and (iii) ongoing research into low-cost polymer formulations, membrane-free or hybrid architectures, and improved electrode materials is still in the development phase for producing a mature technology.

Herin, the main goal is to develop an optimum electrochemical desalination system that integrates a perm-selective membrane with an efficient electrode system for the microfluidic electrochemical desalination. Specifically, polyvinylidene difluoride (PVDF) membrane fabricated via the electrospinning technique [9] and modified with different nano additives, including graphene oxide (GO), activated carbon (AC), and carbon nanotubes (CNTs), is used in combination with a silver/silver chloride electrode in an electrochemical desalination cell. The perm-selective electrospun membrane was evaluated using different characterization methods: scanning electron microscopy (SEM), Fourier transform infrared spectroscopy (FTIR), porosity, and pore size distribution analysis, contact angle, zeta potential, and conductivity measurements, and the results were reported elsewhere [9]. The silver electrode was prepared by coating silver nanoparticles on a carbon cloth, whereas the silver chloride electrode was prepared using electrochemical chlorination conducted using the three-electrode system. The electrodes were analyzed by X-Ray Diffraction (XRD), cyclic voltammetry (CV), and electrochemical impedance spectroscopy (EIS) to analyze the electrodes crystalline structures and stability. The developed membranes and electrodes were integrated with an electrochemical desalination cell to study the performance of the saline water desalination process.

## 2. Experimental Section

### 2.1. Materials

**Figure 1:**
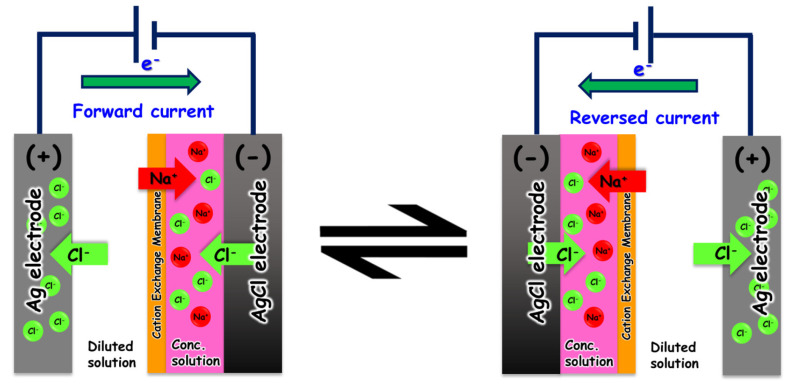
A schematic diagram for the electrochemical desalination setup used in this study.

The schematic diagram of the electrochemical desalination setup used in this study is shown in Figure 1. It consists of two electrodes, separated by a cation exchange membrane, and a power supply. The process begins with introducing saline water with a specific NaCl concentration into the electrochemical cell. Silver and silver chloride electrodes play a crucial battery-type role as they enhance the electrical conductivity and increase the surface area for sodium ion adsorption. A certain potential window is selected using a potentiometer (CHI-Instrument 21.01 CH1842B) for a constant current mode of operation. Chloride ions (Cl^−^) from the saline water move toward the anode, where it is oxidized, transforming the Ag electrode electrochemically to AgCl. The potential window selected is usually within ±1.0 V to avoid water electrolysis. To preserve electrical neutrality, the sodium ion must leave that chamber towards the cathode through the sodium-selective membrane. Ideally, the selective cation-exchange membrane guarantees that sodium ions are the sole ions carried across the membrane to the other chamber where the AgCl electrode is placed. The AgCl electrode in the cathode chamber releases chloride ions to combine with the incoming sodium ions, thus increasing the NaCl concentration in this chamber. Therefore, the water in the anode chamber becomes diluted in NaCl and simultaneously, the water in the cathode chamber becomes more saline. Several parameters affect the performance of the cell, including the power input, the electrodes, the salinity of the processed water, and the membrane nature. At the end of the cycle, when the upper voltage value is reached, and supposedly when the electrodes have been completely inverted (Ag to AgCl and AgCl to Ag), the voltage direction and both chambers’ processes are reversed, thus the name “rocking chair” was poised for this desalination mechanism [10]. The desalination efficiency is tested by calculating the ion removal efficiency, salt adsorption capacity, and specific energy consumption [11].

The membrane’s electrical conductivity, which refers to its ability to conduct electricity, is mainly affected by the ion’s movement. As a result, various factors affect conductivity, including the concentration of ions, the type of the membrane and its resistance, temperature, and thickness [12].

### 2.2. Electrodes Synthesis and Characterization

This section presents the second major component of the study—the electrode system used in the electrochemical desalination process. It details the synthesis of the electrodes using carbon cloth and silver nanoparticles (AgNPs), followed by the silver chlorination procedure carried out with a three-electrode configuration. Additionally, it outlines the methods employed to characterize the electrodes and evaluate their structural, chemical, and electrochemical properties.

The electrochemical desalination process studied in this work follows the rocking chair mechanism that utilizes silver/silver chloride (Ag/AgCl) electrodes. The Ag electrode is prepared from silver nanoparticles (AgNPs) (silver nanopowder < 100 nm particle size, Sigma Aldrich, Dubai, UAE) and carbon cloth (C60, Emirates Scientific and Technical Supplies). Carbon cloth is selected as a substrate to hold the AgNPs because of its large surface area, flexibility, and outstanding conductivity. The AgNPs are dispersed in a mixture of dimethylformamide (DMF) and ethanol using ultrasonication for 3 h. Afterwards, the spray coating method using (KKmoon Dual Action Air Compressor & Spray Gun) was employed to ensure consistent and uniform deposition of AgNPs on the carbon cloth. The coated carbon cloth is then dried overnight at 50 °C. Three different types of C/AgNPs electrodes are prepared, Type-1 (Ag-CC) is the basic C/AgNPs with no additives to serve as a reference for electrochemical performance. Type-2 (X-CC) is a basic C/AgNPs electrode impregnated with Nafion and PVDF as binders, and carbon black as an additive to increase the electrode’s conductivity. Type-3 (Ag-N-CC) is the basic C/AgNPs layered with AgNPs and Nafion without any other modifications.

### 2.3. Chlorination of Silver Electrode

The second electrode required for the electrochemical desalination using the rocking mechanism is silver chloride (AgCl). This electrode is prepared electrochemically from the previously mentioned C/AgCl electrodes. The chlorination reaction is performed using a three-electrode electrochemical cell to oxidize the silver nanoparticles in the C/AgNPs electrode into AgCl nanoparticles. Specifically, the process involves C/AgNPs as a working electrode, a platinum wire as a counter (auxiliary) electrode, and an Ag/AgCl reference electrode immersed in a 1.0 M NaCl electrolytic solution. The system is connected to a potentiostat (CHI-Instrument 21.01 CH1842B) to adjust the potential, allowing the chemical reaction to proceed. When the potential is applied, the chlorine ions in the electrolyte will be attracted to the AgNPs on the working electrode to form AgCl [11] according to the following half-cell oxidation reaction:

(1) $$Ag_{\left(s\right)}+Cl^{-}\to A{gCl}_{\left(s\right)}+e^{-}$$

### 2.4. Electrodes Characterization

The silver and silver chloride electrodes performance is investigated using various characterization methods, including X-Ray Diffraction (XRD) to study the crystalline structure of the electrodes’ materials, cyclic voltammetry (CV) to test the stability and specific capacitance, and electrochemical impedance spectroscopy (EIS) to measure the electrode’s resistance.

#### 2.4.1. X-Ray Diffraction (XRD)

XRD is an important characterization method that provides information about the phase composition and crystalline structure of the electrodes. X-ray waves are scattered by the substrate, generating diffraction patterns. In this study, the XRD peaks (Malvin Panalytical X’Pert3, radius 230 mm, usable range from −40° < 2θ < 160°) are utilized to analyze the presence of different crystalline phases, including carbon, silver, and silver chloride [13].

#### 2.4.2. Cyclic Voltammetry (CV)

CV is one of the most valuable electrochemical characterization techniques because it reliably sheds insight into the electrode’s behaviour, charge-transfer kinetics, electrode stability and durability, and surface reactivity. CV technique was applied to the electrode system used in this study, which includes three electrodes: the working electrode (Ag), reference electrode (Ag/AgCl), and counter (Pt wire) electrode, all submerged in a 0.1 M saline electrolyte and connected to a potentiostat (CHI-Instrument 21.01 CH1842B). A potential is swept with a selected scanning rate (mV/s) over a range of voltage starting from a minimum value to a maximum value and then back to the minimum value, measuring the corresponding current over the two (forward and backward) cycles. A plot of the measured current on the y-axis versus the applied voltage on the x-axis is called a cyclic voltammogram, which reveals important information about the tested electrode and the involved redox reactions. To obtain accurate results, it is crucial to control the reaction kinetics, electrolyte composition, and potential window during the experiment [14].

Specific capacitance (Csp) is an important metric that indicates the electrode’s charge storage capacity by calculating the area under the voltammogram. Csp has an inverse relationship with the scan rate because faster scan rates limit the sufficient time for the ions to diffuse into the surface of the electrode, resulting in low charge storage. Electrodes with high Csp are exhibited with remarkable energy storage and prolonged stability. Csp is calculated using the equation [15]:

(2) $$C_{sp}=\frac{A}{U\;\times \;v{\;\times \;m}_{electrode}}$$

where A is the absolute area of the voltammogram cycle in coulombs (C), U is the potential window in (V), v is the scan rate in (V $s^{-1}$), and $m_{electrode}$ is the active electrode’s mass in (g).

#### 2.4.3. Electrochemical Impedance Spectroscopy (EIS)

EIS helps to identify the charge-transfer resistance, which is a key factor in evaluating the electrode’s efficiency. A low charge-transfer resistance indicates that the electrode experiences slight resistance to electron flow, leading to efficient electron exchange with the electrolyte. The EIS system comprises three-electrode electrochemical system, where the frequency is swept over a certain range. A sinusoidal AC signal is applied to the working electrode. Subsequently, the instrument measures the impedance response of the resistive and reactive components across the frequency range. The resulting data for the electrodes are illustrated in Nyquist plot to present the required resistance values [16].

#### 2.4.4. Electrochemical Setup

**Figure 2:**
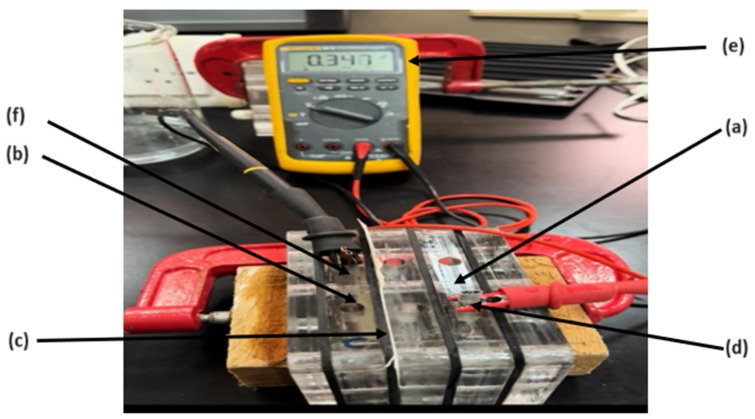
The electrochemical setup used in this study. (a) Anodic chamber, (b) cathodic chamber, (c) CEM, (d/f) Ag/AgCl electrodes, and (e) multimeter.

**Figure 3:**
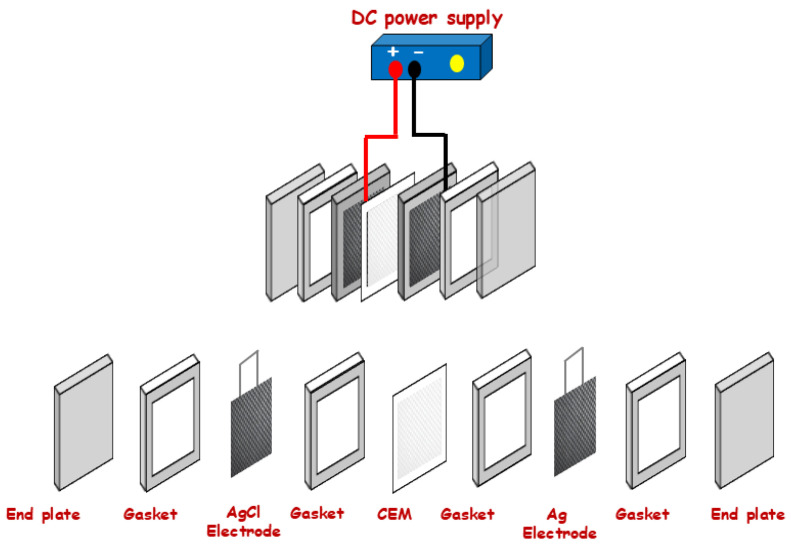
Details of the electrochemical cell components used in this work.

The electrochemical experimental setup consists of two end plates holding the whole cell with Ag and AgCl electrodes in each chamber and the cation exchange membrane (CEM) placed between them. Four gaskets are used to separate the compartments and maintain good cell construction. The different components making up the device are presented in Figure 2 and Figure 3.

Chamber (a) in Figure 2 initially contains the silver (Ag) electrode submerged in saline water with a known initial concentration, while chamber (b) holds the silver chloride (AgCl) electrode immersed in a similar saline solution. Component (c) in the figure is a cation exchange membrane (CEM), which separates the two chambers, to selectively allow the passage of the Na^+^ cations. When a certain low voltage is applied, an electric current is established, driving the movement of ions. The potential of the cell is read by the multimeter (e). The positive Ag electrode (anode) will attract the chlorine ions, which react to form AgCl. The sodium ions will be attracted to the negative AgCl electrode (cathode), leaving chamber (a) to pass through the CEM to the second chamber, where it reacts with the electrode material, forming NaCl. The NaCl concentration in the first chamber will therefore decrease, whereas the NaCl concentration will increase gradually in the other chamber until it reaches a specific level of saturation. After operating the system for a certain time, the process is reversed by switching the direction of the applied voltage with a new saline water. The concentrations of NaCl in both chambers are monitored using a Total Dissolved Solids (TDS) probe, which measures the changes in conductivity at various time intervals. Initially, the experiment was conducted using an available commercial Cation Exchange Membrane CEM (CXM 200 (CMI-7000)) [17] and commercial silver and silver chloride electrodes. The performance of the system was evaluated based on the efficiency of salt removal and the energy consumption for ion transport. The system’s scalability was tested by adjusting the volume of saline water and observing the impact on desalination effectiveness.

## 3. Results and Analysis

### 3.1. Electrodes Characterizations

The AgNPs-based electrodes were fabricated via a spray coating technique, applying the silver nanoparticles onto a carbon cloth substrate. Two separate carbon cloth pieces were coated; one served as the silver electrode, while the other was subsequently chlorinated to yield the silver chloride electrode. The electrochemical properties of these electrodes were then investigated using X-Ray Diffraction (XRD), cyclic voltammetry (CV), and electrochemical impedance spectroscopy (EIS).

**Figure 4:**
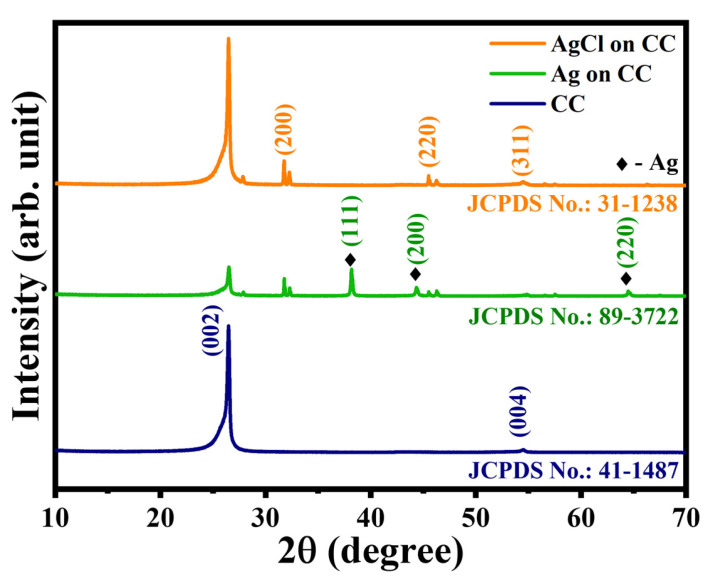
XRD spectra for the bare carbon cloth (CC), Ag-coated carbon cloth (Ag-CC), and AgCl-coated carbon cloth (AgCl-CC) electrodes.

The XRD patterns are recorded from 10 to 70 degrees, and its respective XRD patterns for bare carbon cloth (CC), carbon cloth coated with silver (Ag-CC), and carbon cloth coated with silver chloride (AgCl-CC) are depicted in Figure 4. As shown in this figure the spectra for the CC electrode represent a prominent peak at 2θ ≈ 26°, and a minor peak at 2θ ≈ 54° which agrees with JCPDS card No. 41-1487. These values resemble the values reported in [18]. The (002) plane at 26° reflects the layered graphite structure, whereas the (004) plane at 54° represents crystallinity and arrangements of carbon atoms within the graphite layers. Electrode Ag-CC shows additional peaks due to the silver nanoparticle coatings on the carbon cloth. The peaks at 2θ ≈ 38°, 44°, and 64° corresponding to the face-centred cubic crystal structure of silver at planes (111), (200), and (220) are confirmed for Ag-CC by JCPDS card No. 89-3722 [19]. The peaks observed for the AgCl-CC electrode emphasize the efficient coating of silver chloride on the carbon cloth, specifically at 2θ ≈ 32°, 46°, and 54°. These values validate the silver chloride crystal structure at (200), (220), and (311), planes (JCPDS card No. 31-1238) as previously reported [20].

**Figure 5:**
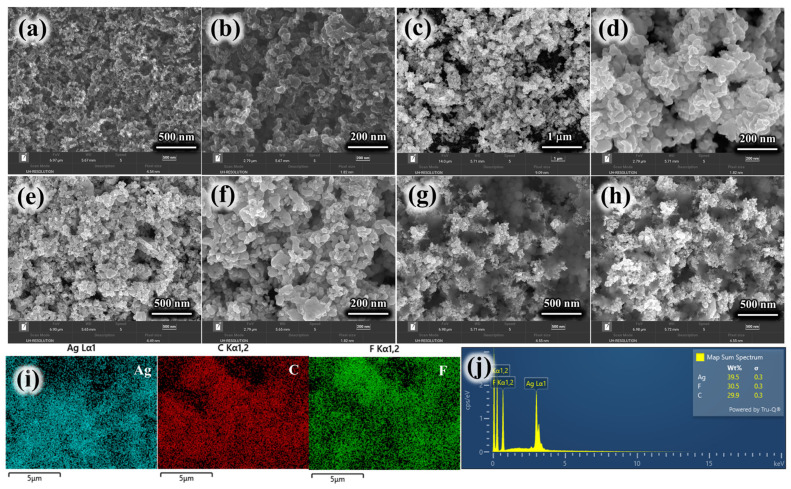
HRSEM analysis (**a**,**b**) CC, (**c**,**d**) Ag-CC, (**e**,**f**) Ag-N-CC, (**g**,**h**) X-CC, (**i**) elemental mapping images, and (**j**) EDX spectra for X-CC electrode.

The morphological characteristics of the fabricated electrodes were systematically investigated through high-resolution scanning electron microscopy (HRSEM). Lower and higher magnified images are shown in Figure 5a,b, respectively. The pristine carbon cloth (CC) exhibits a relatively smooth surface and a uniform carbonaceous layer. This morphology provides an ideal conductive scaffold for subsequent material deposition. Upon incorporation of silver nanoparticles, the Ag-CC electrode, shown in Figure 5c,d, reveals a distinct distribution of Ag NPs anchored onto the carbon cloth surfaces. These nanoparticles adhere strongly to the CC surface, forming a heterogeneous coating that enhances the electrode’s surface roughness and potentially increases its electroactive surface area. A composite mixture consisting of PVDF and Nafion was introduced onto the CC substrate, as presented in Figure 5e,f. The polymeric blend forms a conformal film over the carbon cloth, functioning as both a binder and an ion-conductive medium. Within this matrix, the Ag nanoparticles, carbon black, PVDF, and Nafion are homogeneously intercalated and well-integrated with the CC electrode. Moreover, elemental mapping images in Figure 5i for the X-CC electrode resulting in silver, carbon, and fluorine elements. The corresponding EDX spectra shown in Figure 5j corroborate these findings by confirming the characteristic elemental peaks, thereby supporting the successful deposition and compositional integrity of the electrode materials.

The CV test was conducted for four carbon cloth-based electrodes: bare carbon cloth (CC), Ag-coated carbon cloth (Ag-CC), carbon cloth coated with AgNPs, PVDF, Nafion, and carbon black (X-CC), and carbon cloth coated with AgNPs and Nafion (Ag-N-CC). Each electrode was tested at voltage scanning rates of 10, 20, 30, 50, 100, and 200 mV/s at a potential window from −0.4 to 0.3 V. Figure S1 presents the cyclic voltammograms for the four electrodes mentioned above. The quasi-rectangular shapes of CV curves for all electrodes suggest outstanding double-layer capacitance. The CC electrode exhibited the lowest current response, reflecting the limited capacitive behaviour of the bare CC electrode. However, the Ag-CC electrode displays a modest enhancement. Notably, the X-CC electrode achieved the highest current response, indicating the effective integration of the conductive materials. The Ag-N-CC demonstrated a strong performance, but its current remains slightly below the X-CC current.

**Figure 6:**
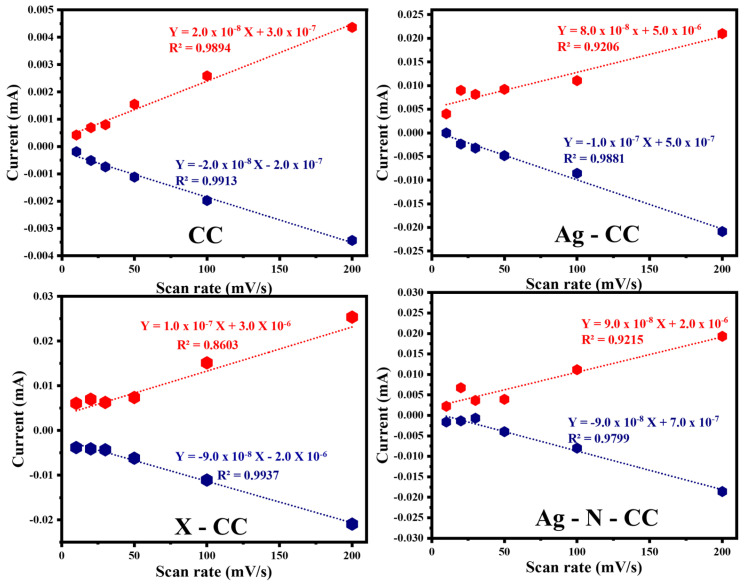
Effects of the scanning rate on the induced current for the different electrodes investigated in this study.

The linear regression plots in Figure 6 illustrate the relationship between anodic and cathodic peak currents and the scan rate for the uncoated and coated carbon cloth electrodes. The X-CC electrode demonstrated the steepest slope, signifying the highest current response and superior capacitive performance. The slope associated with the Ag-N-CC electrode was slightly lower than that associated with the X-CC electrode. In contrast, the CC and the Ag-CC electrodes show lower slopes, indicating weaker electrochemical activity.

**Figure 7:**
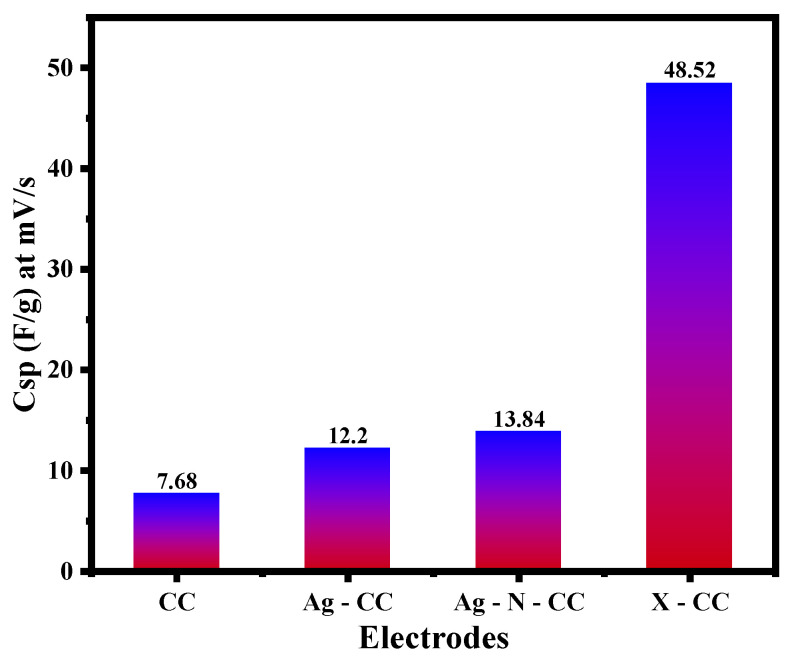
Specific capacitance results for the prepared electrodes at a scan rate of 10 mv/s.

The specific capacitance results are calculated from the cyclic voltammetry data and presented in Figure 7. The results presented in this figure reveal noticeable differences in the electrochemical performance among the four electrodes. The bare CC electrode displayed the lowest specific capacitance across all scan rates, with a maximum value of 7.68 F/g at 10 mV/s. Surface modification of the Ag-CC electrode results in higher specific capacitance compared to the bare CC electrode and reaches a maximum of 12.20 F/g at 10 mV/s. While the Ag-N-CC electrode showed a slightly higher peak with 13.84 F/g at 10 mV/s, the X-CC electrode exhibited exceptional results with a maximum capacitance of 48.52 F/g at the same scanning rate. The various scan rate and area measurement results were tabulated in Table S1.

**Figure 8:**
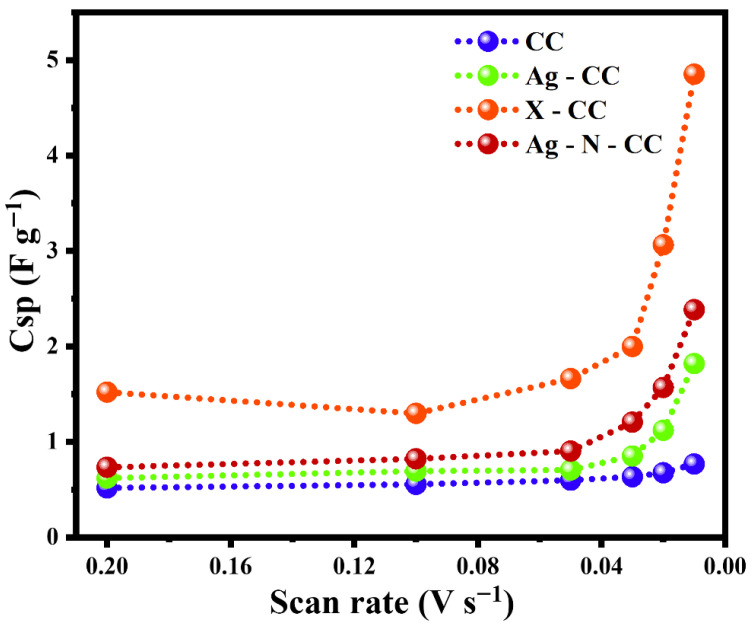
Specific capacitance for the CC, Ag-CC, X-CC, and Ag-N-CC electrodes at all scan rates (0.2–0.01 V/s).

The inverse relationship between the specific capacitance and scan rates for all electrodes is clear in Figure 8 (note the inverted x-axis scale). At high scan rates, there is less time for ions to diffuse into the electrode material, limiting the accessible charge storage sites, whereas at low scan rates, ions penetrate more effectively, leading to increased specific capacitance.

**Figure 9:**
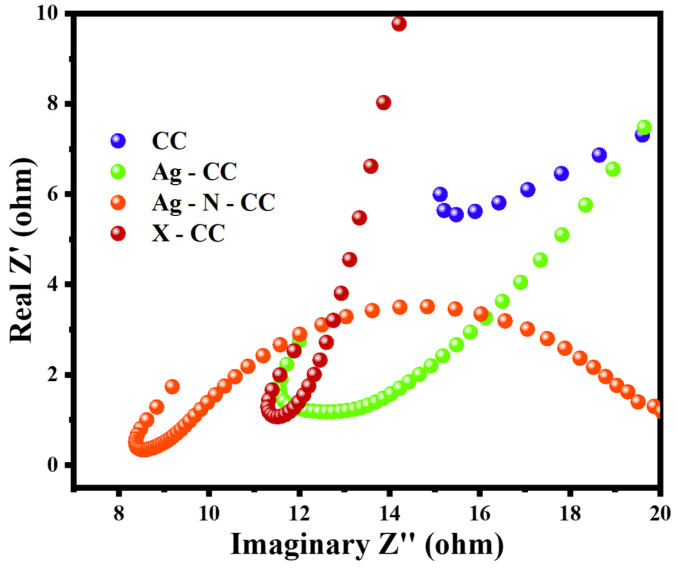
Nyquist Plot for the prepared electrodes.

The EIS test was performed for the prepared electrodes (CC, Ag-CC, Ag-X-CC, and Ag-N-CC) to evaluate their resistance values using the Nyquist plot. The Nyquist plots in Figure 9 provide insights into the frequency-dependent resistance of the prepared electrodes. The semicircle observed in the high-frequency region reflects the charge-transfer resistance at the electrode–electrolyte interface. The CC electrode exhibits the highest resistance (15.6 Ω), indicating poor conductivity, while the X-CC electrode shows the lowest resistance (8.9 Ω), demonstrating enhanced charge-transfer kinetics. The Ag-CC and Ag-N-CC electrodes show intermediate resistances of 13.8 Ω and 11.4 Ω, respectively, highlighting the effect of AgNPs coated on the surface of carbon cloth. In the mid-frequency range, semicircle broadening corresponds to double-layer capacitance and surface heterogeneity. The Ag-modified electrodes show slight broadening due to surface roughness, whereas the X-CC electrode exhibits a compressed semicircle, suggesting a more uniform interface. The low-frequency region shows minimal Warburg-type behaviour, indicating limited diffusion effects. Overall, the improvements in X-CC arise mainly from enhanced high-frequency charge-transfer processes. The combination of AgNPs, PVDF, Nafion, and carbon black creates synergistic effects. These additives improve both electronic and ionic conduction pathways. Consequently, X-CC demonstrates superior conductivity and reduced interfacial resistance. Frequency-specific analysis confirms that electrode modifications effectively optimize electrochemical performance.

### 3.2. Results and Analysis of Electrochemical Desalination Performance

Based on the membrane and the electrode characterization results, the following optimized membranes and electrodes were selected for an electrochemical desalination study using the rocking-chair setup previously described: PVDF/GO, PVDF/CNTs membranes, and the X-CC electrode. The cell was tested using a potentiostat, which performed chronopotentiometry experiments within a potential window of −0.6 to 0.6 V. The electrochemical desalination performance was evaluated at three different levels of current: 1, 3, and 10 mA, with initial synthetic saline water of 15,000 and 30,000 ppm NaCl. The solution was sampled every 15 min to measure its salinity using a Total Dissolved Solids (TDS) conductivity probe.

The experimental programme conducted in this work included one experiment using the Ag/AgCl electrode system without a membrane (pure capacitive deionization experiment) to serve as a reference and five other experiments, each with a different membrane/additive combination. Five experiments involved measuring the salinity-time profiles for a synthetic brine solution of an initial salt concentration of 15,000 ppm at three levels of current (1, 3, 10 mA), and the sixth experiment studied a synthetic brine solution of 30,000 ppm initial salt concentration. For each experiment, three calculated desalination metrics were calculated: the ion removal efficiency, the specific adsorption capacity (SAC) in mg/g, and specific energy consumption (SEC) in kJ/mole. The six experiments conducted are as follows: (1) Ag/AgCl electrodes without a membrane, (2) Ag/AgCl electrodes with a commercial cation exchange membrane, (3) Ag/AgCl electrodes with a pure PVDF membrane with no additive, (4) Ag/AgCl electrodes with PVDF/CNTs, (5) Ag/AgCl electrodes with PVDF/GO at 15,000 ppm initial salinity, and (6) Ag/AgCl electrodes with PVDF/GO at 30,000 ppm initial salinity. The experiments were performed with a potential window of −0.6 to 0.6 V, volume of saline solution in the cell of 200 mL, and mass of each electrode of 1.4702 g. The experimental data are available as a supplement to this paper; however, as an example, we present in Table 1 the experimental data measured, together with the above three desalination metrics, for experiment 1.

In brief, ion removal efficiency describes how efficient the desalination process is in removing specific ions from the NaCl solution contained within the electrochemical cell [11]. The percentage of ion removal efficiency is calculated using Equation (3):

(3) $$(\%)\;=\;\frac{C_{i}-C_{f}}{C_{i}}\times 100$$

where $C_{i}$ is the initial concentration, and $C_{f}$ is the final concentration.

Salt adsorption capacity (SAC) of an adsorbent material is defined as the amount of salt that can be removed by a unit mass of that adsorbent material. In this study, the SAC is calculated using Equation (4) below [11].

(4) $$SAC\;\left(mg\cdot g^{-1}\right)=\left(\frac{{MW}_{NaCl}}{m_{e}}*\Delta C_{i}*V_{cell}\right)$$

where MW_NaCl_ is the molecular weight of sodium chloride, $m_{e}$ is the mass of both electrodes, $V_{cell}$ is the volume of the chamber, $C_{i}$ is the initial concentration, and $\;C_{f}$ is the final concentration.

The specific energy consumption (SEC) represents the amount of energy consumed throughout the desalination process per unit mole of the salt removed as determined using Equation (5) below [11].

(5) $$SEC\;\left(kJ\cdot {mole}^{-1}\right)=\frac{\int_{0}^{t}IVdt}{moles\;of\;salt\;removed}$$

where V is the time-dependent cell voltage, and I is the applied current.

**Figure 10:**
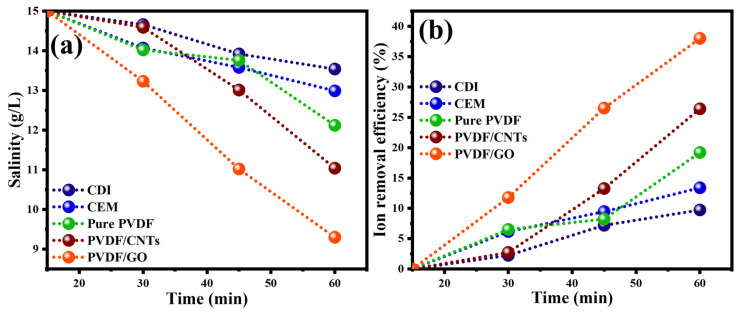
(**a**) Time profile for salinity of the treated prine samples and (**b**) ion removal efficiency for the different membranes at a current level of 10 mA.

Figure 10 shows the time profile for the salinity of the treated brine samples and the percentage ion removal efficiency for the different membranes when integrated with the Ag/AgCl electrochemical desalination setup. The PVDF/GO membrane gave the best performance in terms of salinity reduction; salinity over one hour dropped from 15,000 ppm to 9300 ppm for the 10 mA current level, which is equivalent to an ion removal efficiency of 68% as Figure 10b shows. In our previously published work [9], the surface charges of the prepared membranes with the additives were evaluated by zeta potential analysis after applying a sulfonation process. The zeta potential analysis showed negative values, which correlated with the presence of sulfonic groups on the membrane’s surface, confirming that the resulting membranes were cation-exchange. The PVDF/CNTs membrane comes next in terms of salinity and ion removal efficiency as clear from Figure 10.

**Figure 11:**
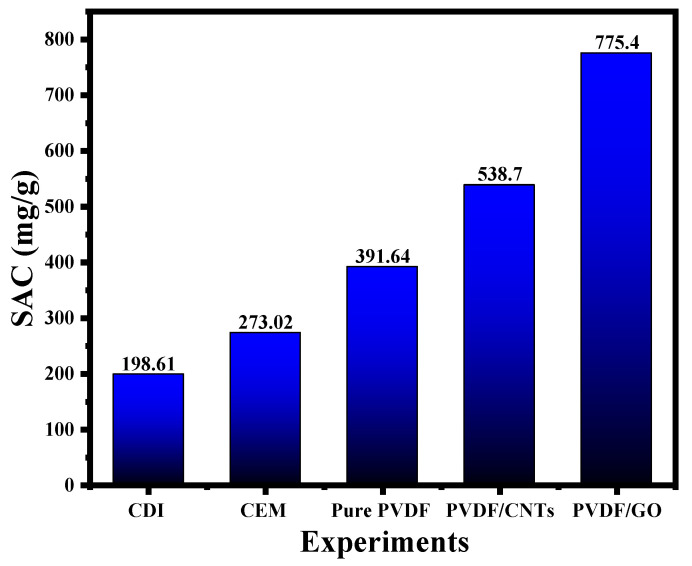
SAC results for the electrochemical desalination experiments conducted in this work.

Figure 11 shows the salt adsorption capacity (SAC) for the different membranes. The SAC for the PVDF/GO membrane is almost four times that of the base case, where no membrane was used in the electrochemical desalination (pure capacitive deionization experiment). When the PVDF membrane was used with no additives, the SAC value reached a value of 391.64 mg/g. However, when the PVDF membrane was used in combination with CNTs, the SAC reached a value of 538.7 mg/g.

**Figure 12:**
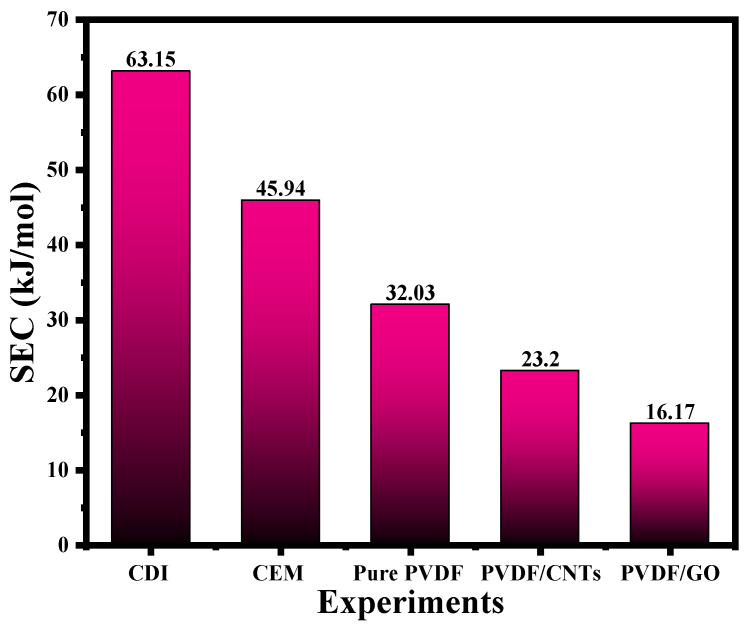
SEC results for the electrochemical desalination experiments conducted in this work.

The specific energy consumption (SEC) metric for the different membrane systems used in this study was calculated according to Equation 5 and are presented in the bar chart shown in Figure 12. The SEC value for the base case experiment, where no membrane is used, is 63.15 kJ/kg as shown in this figure. Employing a cation exchange membrane (CEM) in general leads to a reduction in the SEC. For example, incorporating a commercial CEM reduces the SEC to 73% of that of the base case, whereas with the PDVF membrane, the SEC is reduced by 51%. The reduction in the SEC was also found to depend on the nature of the nano additive incorporated in the membrane. In terms of SEC, the best membrane system was the PVDF/GO, where a reduction of up to 26% of the base case was observed. The results obtained are promising in enhancing the adsorption capacity with minimum cost compared to the current data available in the literature. For example, Haq et al. [21] used polyamine/carbon composites with amino or sulfonic-acid groups as capacitive deionization (CDI) electrodes. The materials exhibited high charge efficiency of 90% and desalination capacity of 17.7 mg·g^−1^ compared to the original polyamine/carbon composites values of 86% and 14.7 mg·g^−1^ [21]. Also, an integrated membrane–electrode prepared by spray-coating a thin layer of an ion exchange polymer on the activated carbon electrode showed a SAC of up to 14–20 mg·g^−1^ [22]. In another study, Hou et al. [23] have reported that carbon aerogel has dual function as an electrical double layer capacitor and as a potential electrode in capacitive deionization processes, where optimal equilibrium electro-sorption capacity of 270.59 mmol/g at 1.2 V was reached.

## 4. Conclusions

This study presents a lab-scale systematic experimental investigation of electrochemical desalination of saline water using an electrospun permselective polyvinylidene difluoride (PVDF) membrane modified with nano additives and integrated with an Ag/AgCl electrode system. The Ag electrode was fabricated by spray-coating silver nanoparticles (AgNPs) onto high surface area carbon cloth (CC). The electrochemical performance of bare CC, Ag-coated CC (Ag-CC), AgNPs blended with Nafion and carbon black (X-CC), and silver/Nafion/carbon cloth (Ag-N-CC) electrodes was evaluated using cyclic voltammetry (CV) and electrochemical impedance spectroscopy (EIS) to assess stability and charge-transfer resistance. Among the tested electrodes, X-CC exhibited the highest current response and the lowest resistance, as confirmed by CV cycling and Nyquist analysis. These electrodes, in combination with the optimal PVDF-based membrane, demonstrated that the PVDF/GO membrane integrated with the Ag/AgCl system delivered the best desalination performance based on calculated desalination metrics, achieving an ion removal efficiency of 68%, a salt adsorption capacity of 775.40 mg·g^−1^, and a specific energy consumption of 16.17 kJ mol^−1^.

## Figures and Tables

**Table 1:** Electrochemical desalination results for three different levels of current using Ag/AgCl electrodes and no membrane.

Current = 1 mA				
Salinity (ppm)	Time (minutes)	Ion Removal %	Salt Adsorption Capacity (mg/g)	Specific Energy Consumption (kJ/mole)
15,000	15	0	95.08	13.19
14,820	30	1.20		
14,672	45	2.19		
14,301	60	4.66		
**Current = 3 mA**				
15,000	15	0	96.58	38.96
14,700	30	2.00		
14,332	45	4.45		
14,290	60	4.73		
**Current = 10 mA**				
15,000	15	0	198.61	63.15
14,664	30	2.24		
13,920	45	7.2		
13,540	60	9.73		

## Data Availability

The data presented in this study are available on request from the corresponding author due to privacy or ethical restrictions.

## Supplementary Materials

The following supporting information can be downloaded at: https://www.mdpi.com/article/10.3390/membranes16020062/s1, Figure S1: CV test for the electrodes: CC, Ag-CC, X-CC, and Ag-N-CC; Table S1: Specific capacitance calculation results; Table S2: Desalination results for experiment.
