# Supplementary material for: Nanomaterial Enhanced PVDF Mixed Matrix Membranes for Microfluidic Electrochemical Desalination
Source: Membranes (Basel). 2026 Feb 2;16(2):62. doi: 10.3390/membranes16020062 (PMC12943106; doi:10.3390/membranes16020062)
Supplement: Supplementary file 1 [file membranes-16-00062-s001.zip › membranes-3982186-supplementary.pdf]

Supplementary Materials

Figure: S1

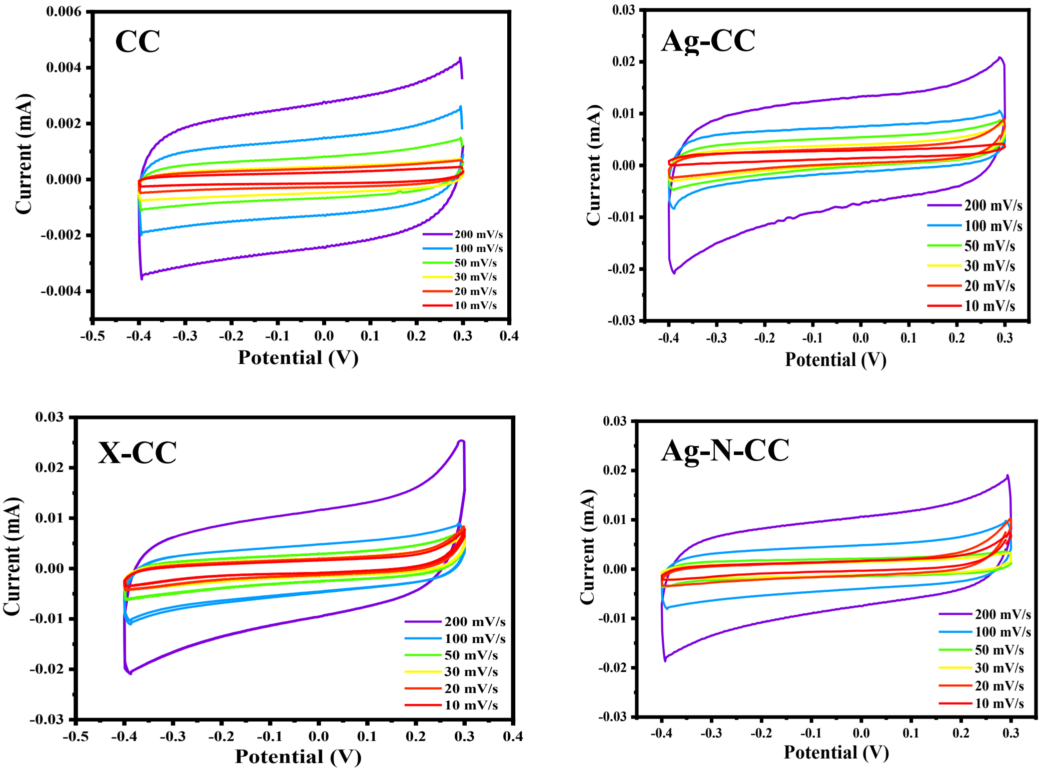

Figure S1: CV test for the electrodes: CC, Ag-CC, X-CC, and Ag-N-CC.

Table S1: Specific capacitance calculation results

	Area (AV)	Scan Rate (V/s)	Mass (g)	Specific Capacitance (F/g)
Bare CC	3.47388	0.2	0.024	5.169
	1.86135	0.1	0.024	5.539
	1.00427	0.05	0.024	5.977
	6.38335	0.03	0.024	6.332
	4.54314	0.02	0.024	6.760
	2.57971	0.01	0.024	7.677
Ag-CC	Area (AV)	Scan Rate (V/s)	Mass (g)	Specific Capacitance (F/g)
	1.47722	0.2	0.064	8.192
	6.24178	0.1	0.064	6.923
	4.08308	0.05	0.064	9.057
	2.84319	0.03	0.064	10.511
	2.1752	0.02	0.064	12.062
	1.09988	0.01	0.064	12.199
X-CC	Area (AV)	Scan Rate (V/s)	Mass (g)	Specific Capacitance (F/g)
	2.84529	0.2	0.0668	15.212
	1.21381	0.1	0.0668	12.979
	7.76951	0.05	0.0668	16.615
	5.60693	0.03	0.0668	19.984
	5.73197	0.02	0.0668	30.645
	4.53747	0.01	0.0668	48.518
Ag-N-CC	Area (AV)	Scan Rate (V/s)	Mass (g)	Specific Capacitance (F/g)
	1.2704	0.2	0.0716	6.336
	6.23828	0.1	0.0716	6.223
	2.52078	0.05	0.0716	5.029
	1.82304	0.03	0.0716	6.062
	2.11875	0.02	0.0716	10.568
	1.38743	0.01	0.0716	13.841

Table S2: Desalination results for experiment

	Salinity (ppm)	Time (min)	Current (mA)	Ion removal efficiency (%)	SAC (mg/g)	SEC (kJ/mole)
Experiment 2: CEM Commercial Cation Exchange Membrane	15000	15	1	0	229.76	5.45
	14533	30	1	3.11		
	13900	45	1	7.33		
	**13311**	60	1	11.26		
	Salinity (ppm)	Time (min)	Current (mA)	Ion removal efficiency (%)	SAC (mg/g)	SEC (kJ/mole)
	15000	15	3	0	267.99	14.04
	14225	30	3	5.17		
	13729	45	3	8.47		
	**13030**	60	3	13.13		
	Salinity (ppm)	Time (min)	Current (mA)	Ion removal efficiency (%)	SAC (mg/g)	SEC (kJ/mole)
	15000	15	10	0	**273.02**	**45.94**
	14075	30	10	6.17		
	13580	45	10	9.47		
